# Supplementary material for: A Critical View of the Application of the APEX Software (Aqueous Photochemistry of Environmentally-Occurring Xenobiotics) to Predict Photoreaction Kinetics in Surface Freshwaters
Source: Molecules. 2019 Dec 18;25(1):9. doi: 10.3390/molecules25010009 (PMC7017383; doi:10.3390/molecules25010009)
Supplement: Supplementary file 1 [file molecules-25-00009-s001.zip › 3.2.4_gcc-4.4.0.zip!/3.2.4_gcc-4.4.0/3.2.4_gcc-4.4.0/doc/octave/html/interpreter/Basic-Usage.html]

Basic Usage - Untitled


Next: Matrix Algebra,
Up: Diagonal and Permutation Matrices


---

### 20.1 Creating and Manipulating Diagonal and Permutation Matrices

A diagonal matrix is defined as a matrix that has zero entries outside the main diagonal;
that is,
`D(i,j) == 0` if `i != j`.
Most often, square diagonal matrices are considered; however, the definition can equally
be applied to nonsquare matrices, in which case we usually speak of a rectangular diagonal
matrix.

A permutation matrix is defined as a square matrix that has a single element equal to unity
in each row and each column; all other elements are zero. That is, there exists a
permutation (vector)
`p` such that `P(i,j) == 1` if `j == p(i)` and
`P(i,j) == 0` otherwise.

Octave provides special treatment of real and complex rectangular diagonal matrices,
as well as permutation matrices. They are stored as special objects, using efficient
storage and algorithms, facilitating writing both readable and efficient matrix algebra
expressions in the Octave language.

- Creating Diagonal Matrices- Creating Permutation Matrices- Explicit and Implicit Conversions
